# Cohort Analysis of Epithelial Cancer Mortality Male-to-Female Sex Ratios in the European Union, USA, and Japan

**DOI:** 10.3390/ijerph17155311

**Published:** 2020-07-23

**Authors:** Greta Carioli, Paola Bertuccio, Fabio Levi, Paolo Boffetta, Eva Negri, Carlo La Vecchia, Matteo Malvezzi

**Affiliations:** 1 Department of Clinical Sciences and Community Health, Universitá degli Studi di Milano, 20133 Milan, Italy; greta.carioli@unimi.it (G.C.); carlo.lavecchia@unimi.it (C.L.V.); 2 Department of Biomedical and Clinical Sciences, Universitá degli Studi di Milano, 20157 Milan, Italy; paola.bertuccio@unimi.it (P.B.); eva.negri@unimi.it (E.N.); 3 Institute of Social and Preventive Medicine (IUMSP), Unisanté, University of Lausanne, CH-1010 Lausanne, Switzerland; fabio.levi@bluewin.ch; 4 Stony Brook Cancer Center, Stony Brook University, Stony Brook, NY 11794, USA; paolo.boffetta@unibo.it; 5 Department of Medical and Surgical Sciences, University of Bologna, 40138 Bologna, Italy

**Keywords:** epithelial cancer, mortality, sex ratios, age period cohort, trends

## Abstract

Objective: To illustrate trends in sex ratios in epithelial cancer mortality in the EU, USA, and Japan, with a focus on age-specific and cohort patterns. Methods: We obtained certified deaths and resident populations from the World Health Organisation for the period of 1970–2014 for the USA, Japan, and the EU for 12 epithelial cancer sites. From these, we calculated both the age-specific and age-standardised male-to-female mortality sex ratios. We applied an age-period-cohort model to the sex ratios in order to disentangle the effects of age, period of death, and birth cohort. Results: Age-standardised mortality sex ratios were found to be unfavourable to males, apart from thyroid cancer. The highest standardised rates were in laryngeal cancer: 7·7 in the 1970s in the USA, 17·4 in the 1980s in the EU, and 16·8 in the 2000s in Japan. Cohort patterns likely to be due to excess smoking (1890 cohort) and drinking (1940 cohort) in men were identified in the USA, and were present but less defined in the EU and Japan for the oral cavity, oesophagus, liver, pancreas, larynx, lung, bladder, and kidney. Conclusion: Mortality sex ratio patterns are partly explained by the differences in exposure to known and avoidable risk factors. These are mostly tobacco, alcohol, and obesity/overweight, as well as other lifestyle-related factors.

## 1. Introduction

A male disadvantage in cancer incidence and mortality has long been documented [1,2]. The main risk factors to which differences in cancer incidence and mortality between sexes have been attributed, aside from well-established lifestyle exposures already known to be strongly associated with sex (e.g., smoking, alcohol consumption, and numerous occupational exposures), are related to carcinogenic susceptibility (including genetics), hormones, anthropometric measures, susceptibility and exposure to infections, and the use of health care services, along with their accessibility [3,4].

Recently, sex ratios and other measures of heterogeneity between the sexes have been studied, underlining both how different sites and cancer histologies can be classified according to their sex-differential aetiologies, and the interest to study these sex differentials from a cohort perspective [3].

Our aim is to update information on sex ratios in epithelial cancer mortality, with a focus on age-specific and cohort patterns, using innovative statistical modelling and descriptive graphical techniques. 

## 2. Materials and Methods 

We obtained official matrices of certified deaths and the resident population from the World Health Organisation (WHO) mortality database (WHOSIS) over the period of 1970–2014 for the United States of America (USA), Japan, and 28 countries of the European Union (EU, as defined in July 2013) excluding Cyprus, due to a lack of data [5]. When pooling data to construct the EU as a whole, if data for a cause or country were missing for a period, we used the nearest available (temporal) data.

All causes of cancer death were recorded according to the 10th Revision of the International Classification of Diseases (ICD) [6]. We considered 12 cancer sites: oral cavity and pharynx (C00–C14), oesophagus (C15), stomach (C16), colorectum (C17–C21, C26), liver (specified as primary) (C22.0–C22.7), pancreas (C25), larynx (C32), lung (C33–C34), skin (including melanoma) (C43–C44), bladder (C67), kidney and other urinary sites (C64–C66, C68), and thyroid cancer (C73).

We computed age-specific rates for each cause of cancer death, sex, five-year age group (from 0–4 to 85+ years, 80+ for the USA) and quinquennium. We computed age-standardized rates per 100,000 men and women at all ages using the direct method, on the basis of the world standard population [7]. We calculated male-to-female rate ratios (RR) for both standardised rates across all ages and for raw age-specific rates between the 30–34 and 85+ (80+ for the USA) age groups. These figures are presented in heatmap tables, with calendar quinquennium along the horizontal axis and age quinquennium along the vertical one; age-standardised RRs across all ages for the corresponding calendar quinquennium are given along the top row. To highlight changes in values, each period and age cell is colour-coded along a gradient colour scale from the lowest (green) to the highest (red) with white as the median, and the scales are table-specific. 

To disentangle the effects of age, period of death, and cohort of birth, we applied an age-period-cohort (APC) model to quinquennial age-standardised rates and RRs from 1970–1974 to 2010–2014 quinquenniums. Age groups were limited to those between 30 (30–34 years) and 84 (80–84 years, 75–79 for the USA) years, to avoid the effects of excessive variation in recent, young cohorts. Cohorts were defined according to their central year of birth, the earliest cohort being that of 1890. We carried out the analysis using the APC R package [8]. Briefly, the age, period, and cohort effects were estimated using a log-linear Poisson model with a likelihood penalizing function. To model RRs, instead of using a log linear Poisson model, a normal model was applied to log transformed RRs [9]. Confidence intervals (CIs) of 95% were calculated with a parametric simulation. The age effects were interpretable as mean age-specific death rates per 100,000 persons over the analysed period, in the case of RRs as the mean age-specific male-to-female RR, while the period and cohort effects are expressed as multiplicative effects in relative terms against their weighted average set to unity [8].

## 3. Results

**Figure 1:**
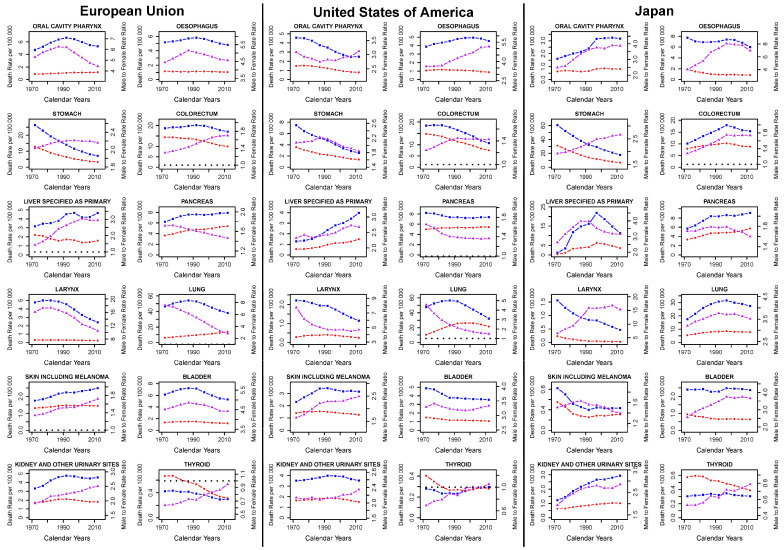
Quinquennial age-standardised mortality rates (blue squares for males, red circles for females) on the left Y-axis, and male-to-female age-standardised sex ratio (purple triangles) on the right Y-axis for major epithelial cancers in the European Union (EU), USA, and Japan, 1970–2014.

Table S1 gives the population age structure by sex for the studied populations as an average number of inhabitants for the first and last quinquenniums considered (1970–1974 and 2010–2014), as well as mean and median ages. Total populations grew and aged over the studied period, and the median age rose 10 years in the EU and USA, and 15 in Japan. Women were more numerous than men (about 5% in the EU and Japan and around 2% in the USA in the most recent quinquennium). Figure 1 shows plots for age-standardised mortality rates for men (blue squares) and women (red circles) at all ages, and their male-to-female RRs (purple triangles) for the studied cancers in the EU, the USA, and Japan. Apart from thyroid cancer, male-to-female RRs were greater than unity (male mortality rates are higher). In the EU, RRs were either descending or stable over the last decade, with the exceptions of skin, kidney, and thyroid cancers. RR patterns for oral, oesophageal, laryngeal, lung, skin, and bladder cancers followed the patterns in male standardised mortality rates across all ages. Conversely, over the most recent quinquenniums in the USA, male-to-female mortality RRs were either rising or stable, with the exceptions of stomach and lung cancers; only stomach and skin cancer RR patterns had any similarity to their male mortality counterparts. In Japan, sex ratio trends for the most recent quinquenniums have been mostly stable in oral, colorectal, laryngeal, and bladder cancers, rising in stomach, kidney, and thyroid cancers, and descending in oesophageal, pancreatic, lung, and skin cancers. Liver cancer RR was descending but reaching an asymptote. Determining whether male or female mortality trends were driving the sex RRs by inspection in Japanese data is more complicated than in the EU and USA. 

**Figure 2:**
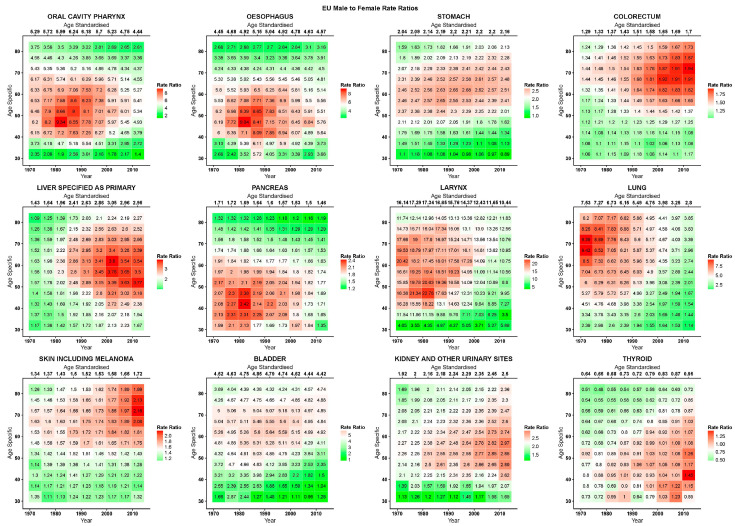
Heatmap tables of age-specific and age-standardised quinquennial male-to-female mortality rate ratios in epithelial cancers in the EU.

**Figure 3:**
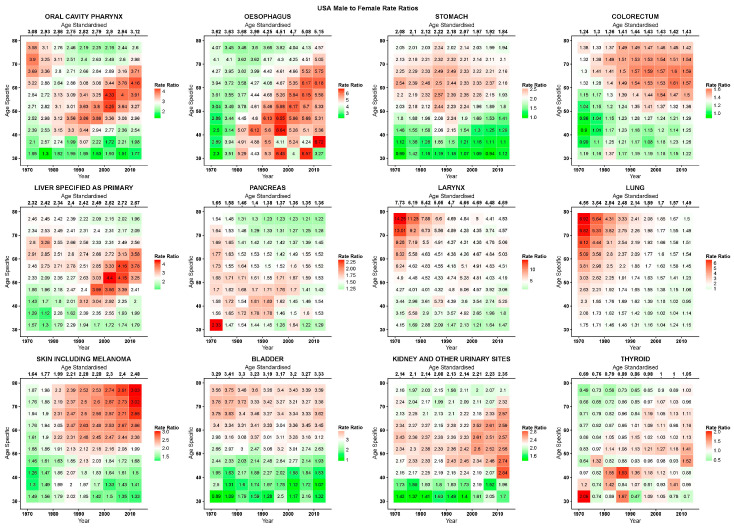
Heatmap tables of age-specific and age-standardised quinquennial male-to-female mortality rate ratios in epithelial cancers in the USA.

**Figure 4:**
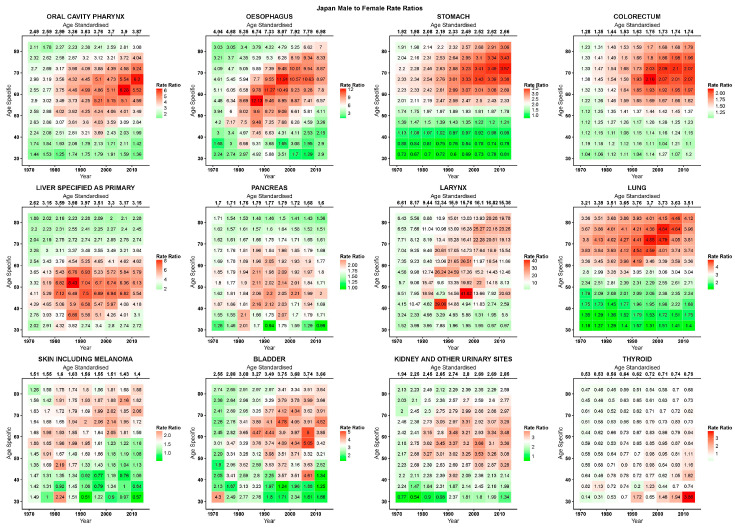
Heatmap tables of age-specific and age-standardised quinquennial male-to-female mortality rate ratios in epithelial cancers in Japan.

Figure 2, Figure 3 and Figure 4 give, for the EU, USA, and Japan respectively, the RR heatmap tables for the studied cancers. The highest and lowest age-standardised RRs were in cancers of the larynx (EU 1980–1984: 17·3; USA 1970–1974: 7·7; Japan 2005–2009:16·8) and thyroid (EU 1970–1974: 0·6; USA 1970–1974: 0·7; Japan 1970–1979:0·5), respectively. The only age-specific RRs under unity were in thyroid cancer, and in the youngest age groups and for most recent years in stomach cancer in the EU and Japan, in under 50 year olds in the early 1970s in colorectal cancer in the USA, and in Japan in the youngest age group after 2000 in skin cancer and before 1990 in kidney cancer. In the EU, oral, oesophageal, laryngeal, and bladder cancers showed peaks in RRs across all ages before the 1990s, and then presented descending values. Inspecting the age-specific RRs for these cancers, we observed high values for young and old ages with peaks at middle age; similarly, RR values were lower at the beginning of the studied period, relatively throughout the age groups, and rose to reach a peak in the 1980s to then descend. Visually, this corresponds to a stronger red area centered on the age period peak (middle-aged, 1980s) that weakened homogeneously along both axes, turning to white and green. Cancers of the colorectum, liver, and skin showed the highest RRs in the upper right quadrant of the tables, corresponding to the more recent years and older age groups, consequently showing rising age-standardised RRs reaching 1·7 for the colorectum and skin and 2·9 for the liver in 2010–2015.

In the USA, oral cavity and pharyngeal, oesophageal, and thyroid cancers showed a cohort peak in age-specific RRs around those born in the 1940s (red diagonal pattern from lower left to upper right of table). Liver and pancreatic cancers also showed this pattern, albeit being slightly shifted towards those born in the 1950s and less evident. Pancreatic cancer also showed high values in age-standardised and age-specific RRs in the earliest periods. A second pattern, most prevalent in lung cancer, but also present in the oral cavity and pharynx, laryngeal, and possibly liver cancers showed its highest values in the oldest cohort (75–79 year olds who died in 1970–1974) centered in 1895 and attenuated moving down the younger cohorts (diagonally downwards, and the bright-red on the right turns to white and green). This pattern was also somewhat visible in bladder cancer. Stomach cancer had a sex ratio peak centered around the age of 60 and the late 1980s. Colorectal and skin cancers both had higher age-specific RRs towards the older age groups and most recent years. Kidney and urinary tract cancers had an age pattern with high RRs between 40 and 60 years of age.

In Japan, cancers of the oral cavity and pharynx, oesophagus, larynx, skin and melanoma, and bladder displayed similar patterns with different intensities: higher male-to-female age-specific RRs were displayed for cohorts born between the late 1930s and 1950s and were visible from the 1980s over the age of 40. Cancers of the stomach and colorectum had similar patterns, with substantially lower male-to-female age-specific RRs at ages under 50. The highest RRs were in those born around the 1940s, mostly after the 1990–1995 quinquennium. Liver and pancreatic cancers had higher age-specific RRs around age 50, starting in the late 1970s/early 1980s, with lower values in the youngest and oldest age groups. Kidney and urinary tract cancers had a similar pattern, with the age peak happening slightly later at 60 years. Lung cancer showed a coherent strong age pattern throughout the studied period, that is, age-specific sex RRs rose with age, particularly after 55 years. However, within this pattern there was also a visible peak in the cohort of those born around 1925. 

**Figure 5:**
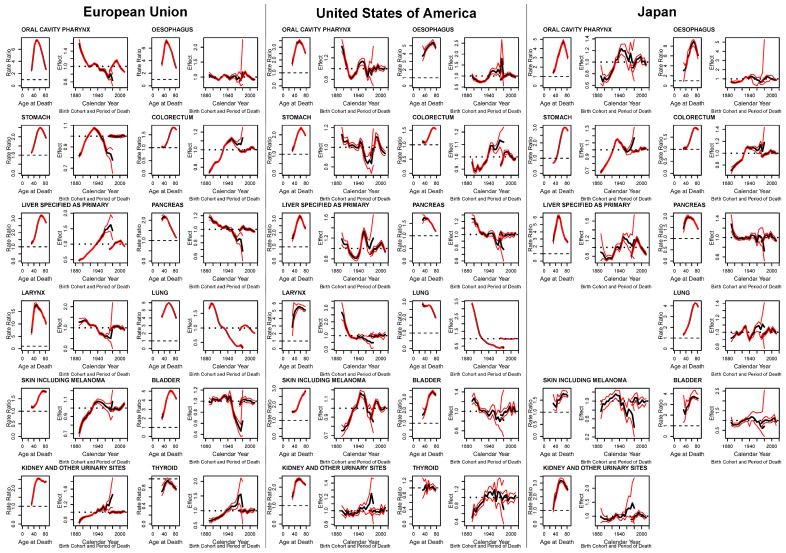
Age-period-cohort analysis of male-to-female sex ratios in major epithelial cancer sites in the EU, USA, and Japan.

APC effects for epithelial cancer mortality male-to-female RR in the EU, USA, and Japan are given in Figure 5 (results for laryngeal and thyroid cancer in Japan are omitted due to excessive variability). Age estimates are expressed as average age-specific male-to-female RRs, while cohort and period estimates are expressed as multiplicative effects relative to the age estimates (estimates are in black, simulated 95% CIs are in red). Age effects prevalently showed a similar pattern with a strong rise, a peak, and then a fall for older ages in age-specific male-to-female RRs. In oral, oesophageal, laryngeal, lung, and thyroid cancers in the EU, and liver and pancreatic cancers in Japan, this pattern showed an inverted “u” or “v”, with RRs in the young and the old age groups showing similarly low values. Cancers of the stomach, colorectum, bladder, and kidney in all the three areas—liver in the EU and USA, skin in the EU and Japan, larynx in the USA, and lung in Japan—showed more of a hook in that the peak is reached at an older age and the RR value does not fall as much. Age effects in male-to-female RRs for the pancreas in the EU and USA, as well as the lung in the USA showed a much earlier peak, followed by a drop that fell lower than where they started. Untangled age-specific RRs for skin cancer in the USA rose throughout the age groups, while they were indeterminate for thyroid cancer in the USA and Japan. 

Cohort effects showed a number of different patterns across cancer sites and countries. Mostly descending patterns through the cohorts were observed in pancreatic cancer in all the three populations, laryngeal and lung cancers in the EU and USA, oral cancer in the EU, and bladder cancer in the USA. Bladder cancer in the EU and stomach cancer in the USA also showed lower effects in recent cohorts, but falls only started after an initial plateau (those born after the mid 1940s). The opposite rising pattern in cohort effects can be seen for kidney cancer in all three populations, and liver and thyroid cancers in the EU. Colorectal cancer also showed a rising trend in cohort effects for the three studied areas; however, these reached a plateau for more recent cohorts. Skin cancer cohort effects in all three areas rose in the 1940s, reached a peak, and then fell; these successive falls were minor in the EU and largest in Japan. Similar trends in cohort effects can be seen for stomach cancer in the EU, thyroid cancer in the USA, and oral cavity and pharynx, and oesophageal cancers in Japan. Oral and oesophageal cancers in the USA, and liver cancer in the USA and Japan started with falling cohort effects up to the cohorts born in the 1930s to then rise up to those born in the 1950s, to then fall again and/or become indeterminate. Cohort effects for stomach and lung cancer in Japan did the opposite, that is, they rose up in the 1930s, fell in the 1950s, and then rose again with widening ICs for the most recent cohorts. Cohort effects for oesophageal cancer in the EU, and laryngeal, bladder, and thyroid cancers in Japan had no discernible pattern or were indeterminate due to excessive variation. Multiplicative period effects were of very modest entity or excessively variable in most cases; however, oral cavity in the EU and Japan, oesophagus, stomach in the USA, liver, pancreas in Japan, lung in EU and Japan, and skin cancer in the USA showed period effects, rising for the earliest quinquenniums with a favourable downturn for the most recent ones.

## 4. Discussion

Cancer incidence and mortality have long been known to be higher in males [1,2]. However, for most cancer sites, the difference between sexes in cancer incidence and mortality has not been satisfactorily explained [3,4]. There is a lack of explicit focus on heterogeneity in cancer studies in favour of aetiology, particularly regarding differences between the sexes. Furthermore, an interest towards assessing cohort effects in sex ratios was expressed [3] and cohort effects in cancer sex ratios have never been comprehensively studied in a modelling context. 

To investigate cohort patterns in cancer mortality male-to-female sex ratios, we used both visual tools on raw age-specific RRs in the form of lexis diagrams enhanced with colour gradients to highlight patterns in male-to-female RRs, and analytic modelling techniques in the form of a modified version of a penalised likelihood APC model [8]. This APC method inherits the limitations of its original implementation—that is, it favours cohort effects due to the higher number of factor levels and younger cohorts tend to be unstable due to the smaller number of at-risk person-years, and hence numbers of events [8]. In addition, the nature of male-to-female RRs also poses limitations: in the case of very low rates (particularly when in females) where the effects this model estimates become unstable (Japanese laringeal and thyroid cancer APC results are omitted for this reason).

Mortality sex ratios are less subject to the influence of changes in diagnosis, coding, and classification of disease, as well as the effects of preventive strategies since they affect both sexes contemporaneously [10]. To minimize the issue of excessive random variation, we limited our study to three geographical areas with very large populations (greater than 100 million) and high-quality mortality data available from the WHO database. However, in interpreting the results from this analysis, the differences within the EU and the US populations must be considered, particularly those between western and northern EU countries and eastern ex-non-market-economy ones. [11,12].

In this work, we analysed epithelial cell cancers and expected to observe patterns in male-to-female RRs corresponding to differences in risk-factor exposures [13].

The more immediately visible patterns in male-to-female RRs were related to smoking and alcohol consumption. The two patterns were particularly distinctly visible in the USA. The pattern due to smoking was clearly defined with a peak in the oldest observed cohort, as seen in lung cancer, for which the attributable fraction due to smoking is about 85% [14]. While the cancer site with the highest attributable fraction due to alcohol is oesophageal cancer (50% in men and 30% women [15]), here, the peak difference is also clear, with a well-defined cohort pattern with a peak for those born around 1940. This cohort also corresponds to that with the greatest difference in obesity prevalence (a recognised risk-factor for oesophageal adenocarcinoma) in the USA, while in the EU, the distribution of obesity prevalence is more heterogeneous [16,17]. Thus, determining which factor is at play when both are associated is difficult. The patterns from alcohol and tobacco, and their combination, according to their respective and combined attributable fractions, can largely explain the differences between the sexes for cancers of the oral cavity and pharynx, oesophagus, larynx, and lung cancer; and to a smaller degree, colorectal, liver, pancreatic, bladder, and kidney cancers [18]. 

Similar patterns are present in the EU and Japan too; however, they are harder to identify and separate. The influence of differences in smoking habits are less evident in the EU in head and neck cancers compared to those in alcohol consumption, while in Japan, they overlap somewhat. Another set of cancer risk factors that are susceptible to sex differentials are infectious diseases, which do not only have different prevalences, but may also have prognostic differences [19,20]. Stomach cancer, for example, while associated with smoking, is strongly associated to *Helicobacter pylori (H. pylori*), diet, and obesity; in Japan, the pattern does have some common traits to those seen in lung cancer, while in the USA and the EU, there is no such similarity. Hence, the pattern in the USA and the EU is more related to differences in *H. Pylori* infection, which is more common in men [20] and more probably due to lifestyle habits throughout successive cohorts [21]. In general, considering that gastric mortality is falling for both sexes in all three populations, whether the sex RR rises or falls depends on the relative speeds at which male and female mortality rates fall in each country. The main risk factors for liver cancer are hepatitis B (HBV) and hepatitis C (HCV), for which women are known to be more resistant to infection, and possibly progression [19]. Particularly for Japan (the high-income country with the highest rate of HCV infection and liver cancer [22]), control of these factors is likely to have determined recent favourable trends in mortality for both sexes and in the sex RR along both the period and cohort time-dimension. The EU’s heterogeneous population probably renders the patterns less defined: HCV being the main risk factor in southern Europe, while in central and northern Europe, heavy drinking is an issue, as well as different timeframes of application of HBV primary prevention [23]. Differently from Japan and the EU, in the USA, patterns recalling the alcohol-like pattern in oesophageal cancer and, albeit less evidently, the tobacco-like pattern from lung cancer are both visible; therefore, in spite of diabetes and obesity being the major risk factors in the USA, heavy alcohol consumption and smoking are likely the strongest differentiators of mortality between the sexes. Pancreatic cancer also has its main known modifiable risk factor in smoking; however, the observed age-specific RR patterns do not appear to have much in common with those seen in lung cancer. The EU and the USA show similar cohort effect patterns to lung cancer ones, but the USA also has the pattern seen for alcohol. This is possibly due to different attributable fractions and latency in cancer onset; other modifiable factors are obesity, diabetes, and heavy drinking [24]. 

The common pattern seen in all three countries for colorectal cancer male-to-female mortality RRs, as well as its being related to risk factors such as alcohol, tobacco, obesity, and sedentariness, and protection from oral contraceptive and hormonal replacement therapy use in women, could also be related to differences in adherence to screening programs, since the ages and periods affected coincide with their period and age of application [25,26]. 

Skin cancer and melanoma are related to ultra-violet radiation exposure; however, due to the excess mortality in men emerging in more recent years and only for older ages, the more accredited explanations for this pattern lie with greater at-risk behaviours in males, including exposure (possibly occupational as well), lack of protection (sun-screen use), and lack of awareness, that is, failure to recognise cancerous lesions, and less frequent contact with medical personnel [27]. However, the lack of, or an inverse pattern at ages under 40 years points to other factors as well. 

Bladder cancer’s strongest recognized risk factor is smoking, and this pattern is somewhat visible in all three areas; occupational exposures which are more prevalent in males also account for the differences [28]. 

The main risk factors for kidney cancer are obesity, smoking, and hypertension; however, the relation of these risk factors to the observed patterns in male-to-female RR is difficult to gauge [29]. 

Thyroid cancer was the only cancer considered that presented a female excess in mortality; however, the fall in mortality from this cancer in both sexes brought the RR back to unity [30]. 

The decreasing RRs in older age groups were observed for most cancers in both the age-specific RRs and the APC analysis, they substantiate the hormonal hypothesis when these falls are seen after middle age, as seen for oral cancers [3]. However, when this fall happens at older age groups, a healthy survivor effect undifferentiated by gender could also be at play.

Genes are also thought to play a role in the sex differences in cancerogenesis, and some sex-specific genetic associations to select tumor sites have been documented; however, the effects of these associations are not satisfactorily quantified, and should be stable in time and not display patterns along the period and cohort time dimensions [31].

## 5. Conclusions

In conclusion, the observed period and cohort patterns in male-to-female cancer mortality RRs are at least partly explained by the differences in exposure to known avoidable risk factors. These are mostly tobacco, alcohol, and obesity/overweight, HCV, and HBV also playing a role in liver cancer. These results underscore the importance of tobacco and alcohol abuse prevention and the promotion of healthy lifestyles, including diet and physical exercise in general.

## Supplementary Materials

The following are available online at https://www.mdpi.com/1660-4601/17/15/5311/s1, Table S1: Average populations over the 1970–1974 and 2010–2014 quinquenniums by 5 year age groups and sex with total population, mean and median ages, for the EU, the USA and Japan.
