# Supplementary material for: Cohort Analysis of Epithelial Cancer Mortality Male-to-Female Sex Ratios in the European Union, USA, and Japan
Source: Int J Environ Res Public Health. 2020 Jul 23;17(15):5311. doi: 10.3390/ijerph17155311 (PMC7432705; doi:10.3390/ijerph17155311)
Supplement: Supplementary file 1 [file ijerph-17-05311-s001.pdf]

Supplementary Table 1. Average populations over the 1970-74 and 2010-14 quinquenniums by 5 year age groups and sex with total population, mean and median ages, for the EU, the USA and Japan.

	EU 1970-74		EU 2010-14		US 1970-74		US 2010-14		Japan 1970-74		Japan 2010-14	
Age	Male	Female	Male	Female	Male	Female	Male	Female	Male	Female	Male	Female
0-4	17885836	17024831	13520086	12840153	8672646	8294983	10205105	9781895	4874720	4622500	2671232	2544660
5-9	18504905	17668076	13337586	12666580	9863257	9576607	10436759	10023170	4267320	4082480	2757763	2630039
10-14	18499134	17669291	13375225	12708356	10720667	10316697	10520363	10027994	4030200	3871060	2969956	2827499
15-19	17902774	17195211	14148321	13433247	10344552	9969686	11156151	10515131	4273460	4151420	3069277	2913843
20-24	17643282	17051740	15695697	15106390	8969682	9119783	11448290	10807518	5240920	5250080	3132494	2976082
25-29	15453630	14940164	16425466	16063692	7676688	7714661	10998720	10630224	4583920	4650500	3483190	3343943
30-34	14959239	14546859	17261456	16968823	6503427	6351955	10486224	10278775	4346360	4398980	3914406	3779386
35-39	14848066	14595273	17820994	17543268	5877740	5753340	10118127	10022245	4171600	4155380	4670133	4514476
40-44	14418538	14448786	18667085	18483805	6021707	5983502	10493947	10353945	3847080	3842080	4695370	4561751
45-49	13591182	14577292	18714141	18714518	6200503	6308687	11068631	11010169	3102380	3403160	4099878	4028549
50-54	11275298	12998737	17557513	17916104	5773262	5995726	11321903	11487111	2249980	2850420	3815672	3797791
55-59	9731226	11480299	16052256	16840641	5066075	5445805	10179205	10591300	2036940	2442200	3976908	4027703
60-64	10501538	12846145	14659265	15826948	4329391	4995885	8540132	9209951	1833000	2141780	4842754	5025961
65-69	8723562	11305575	11893958	13362729	3342844	4058691	6497223	7312330	1434660	1651280	4027757	4366487
70-74	6222382	9085978	10050256	12180875	2380585	3194721	4644807	5398218	1045060	1285680	3435468	3956210
75-79	3582563	6301766	7816116	10626835	1666095	2542751	3356428	4185055	591760	819620	2702834	3466811
80-84	1845749	3645084	5196080	8367542	1507798	2691203	4349201	7273864	264580	441320	1820238	2792617
85+	942156	2100246	3520584	8030694					101180	228840	1230214	3061994
Total	216531060	229481353	245712085	257681200	104916919	108314683	155821216	158908895	52295120	54288780	61315544	64615802
Total Male and Female		446012413		503393285		213231602		314730111		106583900		125931346
Mean Age	33	36	40	43	31	34	37	39	31	33	44	48
Median Age	32	32	42	42	27	27	37	37	27	32	42	47
